# The influence of the literacy environment on children’s writing development in Chinese

**DOI:** 10.3389/fpsyg.2022.1010471

**Published:** 2022-10-13

**Authors:** Mengmeng Su, Yi Fan, Jifeng Wu, Bingyan Qiao, Wei Zhou

**Affiliations:** ^1^College of Elementary Education, Capital Normal University, Beijing, China; ^2^Beijing Key Laboratory of Learning and Cognition, School of Psychology, Capital Normal University, Beijing, China; ^3^College of International Education, Capital Normal University, Beijing, China

**Keywords:** writing development, reading, home literacy environment, primary school, Chinese

## Abstract

The present study investigated the influence of literacy environment on the performance of writing narratives for primary school students. Two hundred and fifty Chinese children participated in this study. There were 146 third graders (71 boys and 75 girls) and 104 fifth graders (53 boys and 51 girls). Results showed that children’s writing abilities differed at the word level and sentence level between third grade and fifth grade. Formal literacy experience (parent teaching of characters) predicted the writing performance of third graders, while informal literacy experience (the visiting frequency of various places) predicted the writing performance of fifth graders. After controlling the effect of reading efficiency on the writing skills, the prediction of formal and informal literacy experiences on the writing performance remained. The results suggest the importance of formal and informal literacy experiences on the writing development of primary school students.

## Introduction

Writing composition is an essential and challenging part in children’s literacy acquisition. As children have mastered how to speak and read, they start to learn writing composition, which is an important skill for learning and working in their later life (Abbott et al., 2010). However, writing as a focus of research has been neglected relative to reading and oral language in recent decades (Miller and McCardle, 2011). Until now, the majority of writing research is in alphabetic languages, whereas writing research in Chinese is scarce. To contribute to expanding knowledge of writing beyond alphabetic languages, the present study focused on the writing skills of Chinese students. In particular, there is still a lack of investigation on the effect of family and social contexts that writing occurs within. The present study therefore seeks to investigate the influence of home literacy environment (HLE) on the performance of Chinese writing for primary school students.

The most influential model of writing to date was proposed by Hayes and Flower (1980) and revised by Hayes (1996). In this model, writing comprises four processes, including planning, translation, reviewing, and revising (Hayes and Flower, 1980; Hayes, 1996). Subsequently, Berninger et al. (2002) proposed a simple view of writing model. In this model, translation is the most important process in writing and it could be modeled as (a) text generation component at different levels of language (word, sentence, and text) and (b) transcription component (handwriting and spelling). According to this model, the most important goal of the developing writer is the text generation component at different levels of language (word, sentence, and text) (Berninger et al., 2002). Assessing children’s writing at different levels of language can localize children’s strengths and weaknesses at the word, sentence, and text levels, thus making further instruction and intervention more targeted (Wagner et al., 2011).

A large number of studies in alphabetic languages have focused on the generation component and measured children’s writing at different levels of language (Whitaker et al., 1994; Berninger et al., 2002; Abbott et al., 2010; Kim et al., 2011; Wagner et al., 2011; Williams and Larkin, 2013; Drijbooms et al., 2015). Recent studies have investigated the development of writing abilities among primary school children at different levels of writing (Puranik et al., 2008; Wagner et al., 2011; Koutsoftas, 2018). Koutsoftas (2018) measured 4^th^ and 6^th^ graders’ writing at the word level and sentence level. Results showed that there was a significant difference at the sentence level, while no significant difference was found at the word level between 4^th^ and 6^th^ graders. In another study, researchers found that there were significant differences between 3^rd^ and 5^th^ graders’ writing at both the word and sentence levels (Puranik et al., 2008). Regarding the text level of writing, a previous study reported no significant difference among 4^th^, 5^th^, and 6^th^ graders (Whitaker et al., 1994). These studies showed various developmental characteristics of the writing at different levels of language.

In Chinese, the basic units of writing are characters, which are more visually complex than alphabetic letters and represent word or morpheme rather than having a grapheme-phoneme correspondence (Lui et al., 2010). Compared with alphabetic language, Chinese has much more homophones (Shu et al., 2003). Consequently, a large number of characters refer to the same syllable. In addition, the grammar and syntax in Chinese are sometimes more ambiguous and difficult to comprehend, because of the free-flowing punctuation and the frequent omission of major sentence components (Yan et al., 2012). Due to those properties, the literacy development in Chinese relies heavily on character learning (e.g., the learning of visual form and the arbitrary connection between visual form and pronunciation) and semantics learning (e.g., the learning of vocabulary and world knowledge) in surrounding context. These learning modalities emerge at home and gradually extend to family activities in other places when parents try to provide a favorable and rich learning environment for their children. Therefore, HLE may play an indispensable role in Chinese children’s literacy development (Shu et al., 2002; Su et al., 2017).

According to educational practices, the development of Chinese primary school children’s writing has been divided into three periods: transforming oral language into writing period (Grade 1, 2), transition period (Grade 3, 4), and preliminary writing period (Grade 5, 6) (Zhu, 1990). The teaching emphasis of the first period is the transcription skills and basic rules of writing. From 3^rd^ grade on, children develop their narrative writing by text reading (Yan et al., 2012; Guan et al., 2014). The teaching emphasis turn out to be improving writing through reading practices (Zhu, 1990). Given the uniqueness of the Chinese writing system, the developmental pattern of Chinese writing has important implications for the formulation of universal theories on writing development across different orthographies.

Compared with ample studies in alphabetic languages, studies in Chinese writing were relatively few and limited to the transcription component (handwriting and spelling) of writing (Tan et al., 2005; Guan et al., 2011). Studies investigating text generation component in Chinese were rare and they did not assess writing according to the levels of language (Guan et al., 2014; Yeung et al., 2017). For instance, Guan et al. (2014) measured Chinese children’s narrative writing in Grades 4, 5, and 6. Children’s response was scored by two research assistants according to three aspects, including expression, content, and commentary. There were significant grade differences among 4^th^, 5^th^, and 6^th^ graders, while no significant grade difference was found between 3^rd^ and 5^th^ graders in another study of narrative writing, in which writing of the children were rated according to four aspects consisting of content, vocabulary, sentence structure, and organization (Yeung et al., 2017). The inconsistency of previous studies may be due to different measures used to assess the writing or the differences in the grade levels of the participants. Moreover, in the previous Chinese studies, a general score of the narrative writing was given to each child. However, a general score could not reflect the specific property of writing. It is essential to explore the developmental characteristic of Chinese writing, using more specific writing indices, such as indices for different levels (word, sentence, and text) of writing.

More recently, theories of writing have emphasized that writing is a communicative act that the individual interacts with the external environment to accomplish challenging writing tasks in the learning environment (Singer and Bashir, 2004; Berninger and Winn, 2006; Berninger and Chanquoy, 2012). As the foundation of writing, emergent literacy development is rooted in communication between people and their environments, which first emerge at home and gradually expand to social context (Vygotsky, 1978; Teale and Sulzby, 1986). Therefore, HLE may play an important role in writing development. To describe the nature of the HLE, Sénéchal and LeFevre (2002) proposed the home literacy model. According to this model, there are two types of home literacy experiences; namely, formal and informal literacy experiences. Formal literacy experience (code-related) engages children directly with print through activities such as parent teaching of reading or spelling. In contrast, informal literacy experience (meaning-related) exposes children to print incidentally through activities such as shared book reading or visit of library (Sénéchal and LeFevre, 2002).

Several studies have found the effects of formal literacy experiences on children’s reading development (Burgess et al., 2002; Manolitsis et al., 2013; Su et al., 2017; Silinskas et al., 2020). For instance, formal literacy experiences indexed by the onset age of parent teaching of reading has been found to be associated with children’s reading in both alphabetic and Chinese languages (Sénéchal et al., 1996; Burgess et al., 2002; Su et al., 2017). In a 11-year longitudinal study, researchers reported that the onset age of parent teaching characters was a significant predictor for reading fluency and reading comprehension for fifth graders (Su et al., 2017). In a recent neuroimaging study, researchers also found that the onset age of parent teaching was related to the property of the left arcuate fasciculus (important brain structure supporting language and reading), even statistically controlling for socioeconomic status (SES), access to print and long-term vocabulary development (Su et al., 2020). Studies from both behavioral and brain levels highlight the essential role of the onset age of parent teaching in the literacy development. Moreover, previous studies have found that the onset age of parent teaching of reading, which attempt to get a cumulative amount of print exposure, were predictive of developmental outcomes than questions that are designed to assess current shared reading practices (DeBaryshe, 1995; Sénéchal et al., 1996; Burgess, 1997). However, until now, the relationship between this variable and writing composition is unclear. Therefore, in the present study, we chose the onset age of parent teaching characters as a representative for the formal literacy experience measure and investigated its association with writing composition.

Regarding informal literacy experience, studies have reported that there is an association between shared-book reading and children’s emergent literacy development (e.g., Manolitsis et al., 2013; Khanolainen et al., 2020) and that the educational outings (i.e., visit of various educational locations) are correlated with primary school children’s academic achievement, including reading, math and science (Coley et al., 2020). As children enter primary school, they gradually acquire the independent reading skill, the frequency of shared-book reading or literacy games may decrease compared with preschool children (Sénéchal and LeFevre, 2014; Inoue et al., 2018). Indeed, researchers did not find any correlation between shared book reading and primary school children’s reading from Grade 1 to Grade 4 (Georgiou et al., 2021). In a recent study, researchers found that the visit of various educational locations (e.g., science center, library, bookstore, art gallery, aquarium, museum, and tourist attraction) was correlated with primary school children’s reading performance (Coley et al., 2020). This study highlights the importance of the educational outings for the primary school children, which extends the informal literacy experience to broad settings outside the home and school. Therefore, we followed Coley et al.’s (2020) study and selected the visiting frequency of various educational locations as items for the informal literacy experience. These visiting experiences may promote children’s vocabulary and world knowledge development by joyous exploration, which may further improve children’s writing development (Dunsmuir and Blatchford, 2004; Kim et al., 2011; Wang, 2017).

Studies have found close relationship between the three levels of writing and reading abilities. For instance, single word reading ability was primarily linked to writing at word level (e.g., writing fluency and spelling), while reading comprehension skills were related to writing at both the word level and the compositional level (e.g., the quality of written content, Berninger et al., 2002; Abbott et al., 2010; Kim et al., 2011; Williams and Larkin, 2013). Considering the tight relationship between reading and writing (Tan et al., 2005; Williams and Larkin, 2013), the connections between HLE and reading could transfer to writing, which may deepen our understanding on the relationship between reading and writing.

However, compared with the abundant evidence on the importance of HLE in reading development, studies exploring the relationship between HLE and writing development were relatively scarce and limited to relatively lower-level skill such as spelling (Sénéchal, 2006; Niklas and Schneider, 2013). For example, Niklas and Schneider (2013) found that HLE (a combination of formal and informal HLE) predicted children’s spelling ability at the end of first grade. Sénéchal (2006) reported that formal literacy experience (indexed by parent teaching of reading) had an indirect effect on children’s spelling ability in the first grade (*via* phonological awareness) and fourth grade (*via* word reading). Until now, little is known about the association between HLE and the writing composition.

In summary, to contribute to expanding knowledge of writing beyond alphabetic languages, the present study investigates on written compositions provided by students in China. Specific research questions were as follows:

1. Assessing Chinese children’s narrative writing according to different levels of language (word, sentence, and text) and investigating the developmental characteristics of Chinese writing in intermediate grade writers (3^rd^ grade vs. 5^th^ grade).
2. Investigating the influence of different aspects of HLE (formal vs. informal) on the three levels (word, sentence, and text) of Chinese writing and comparing the grade difference of the associations between 3^rd^ grade and 5^th^ grade.

We expected that there were significant differences between 3^rd^ and 5^th^ graders on the word, sentence and text levels of narrative writing. Specifically, 5^th^ graders performed better than the 3^rd^ graders on each level of writing (Guan et al., 2014). Furthermore, the roles of formal and informal literacy experience for writing may be different in grades 3 and 5. Specifically, formal literacy experience indexed by parent teaching might play more important role in the word level of writing in the lower graders, while informal literacy experience indexed by various visiting experiences might play more important role in the higher level of writing in the upper graders (McCutchen, 1986; Sénéchal, 2006; Su et al., 2017).

Previous studies have also found that females outperformed males on the narrative writing tasks (U.S. Department of Education, 2008; Troia et al., 2013), but potential differences between females and males in different levels of writing (word level, sentence level, and text level) for the 3^rd^ and 5^th^ graders have not been investigated. Thus, we added sex as a variable of interest in the present study. Moreover, as SES is closely related to HLE in a variety of literacy-related studies (e.g., Sénéchal, 2006; Su et al., 2017), we control this variable in the subsequent regression analyses. Finally, studies have found tight relationship between the three levels of writing and reading abilities (Berninger et al., 2002; Abbott et al., 2010; Kim et al., 2011; Williams and Larkin, 2013). In order to examine the specific association between HLE and writing composition, we added a reading efficiency task (a combination of word reading and reading comprehension) as a control variable in the present study.

## Materials and methods

### Participants

Two hundred and fifty Chinese children participated in this study. They came from three primary schools of Haidian District in Beijing. In each school, we randomly selected two classes (one from third grade and one from fifth grade). Finally, 146 third graders (*M*_*age*_ = 9.1 years, *SD* = 0.5) and 104 fifth graders (*M*_*age*_ = 11.1 years, *SD* = 0.3) participated in this study. The sex ratio (boys/girls) for the third graders and fifth graders were 71/75 and 53/51 respectively. There were no significant differences in the sex percentage between the two groups of children (all *ps* > 0.05). The participants were all native Mandarin speakers with a wide range of reading skills (see Table 1, scores on the reading efficiency task). As they were from the same district, there were nearby opportunities for outside-of-home and school visits (libraries, museums etc.) for all participants. They had normal or corrected-to-normal vision with no history of neurological abnormalities, head injury, or intellectual disability. Family SES was collected by the index of parental education. The overall parent education of the present study was in an upper-middle level (*M*_*edu*_ = 5.5, *Range* = 1–8). Therefore, the sampling of the present study may reflect the situation of upper-middle-class families of China. Informed written consent of the present study was obtained from both the parents and their children.

**TABLE 1:** Scores on the writing, reading, and home literacy environment (HLE) between the two grades.

					Grade 3	Grade 5	Comparisons		
							
Measures	Mean (S.D.)	Skewness	Kurtosis	Reliability	Mean (S.D.)	Mean (S.D.)	t	*P*	Cohen’s d
*Writing composition*									
Word token (WL-1)*	99.2 (38.8)	0.14	−0.143	0.999	86.6 (31.2)	116.8 (41.6)	−6.254	<0.001	0.821
Word type (WL-2)	62.2 (23.2)	0.212	−0.038	0.998	54.4 (17.6)	73.2 (25.7)	−6.472	<0.001	0.854
Sentence premodifier number (SL-1)	7.9 (5.2)	1.03	1.613	0.999	7.0 (4.2)	9.3 (6.1)	−3.346	0.001	0.439
Sentence premodifier mean length (SL-2)	3.2 (1.2)	0.252	0.829	0.99	3.1 (1.2)	3.2 (1.2)	−0.743	0.458	0.083
Text content quality (TL)	13.6 (4.4)	−0.239	−0.122	0.768	13.3 (3.8)	14.0 (5.0)	−1.211	0.227	0.158
*Reading efficiency*									
Sentence reading efficiency (Reading)	314.5 (149.6)	0.777	0.589	0.97	267.2 (144.4)	381.0 (130.9)	−6.38	<0.001	0.826
*Home literacy environment*									
Onset age of parent teaching (Formal)	4.2 (1.3)	−0.357	−0.703	–	4.1 (1.3)	4.4 (1.3)	−1.735	0.084	0.231
Visiting frequency of places (Informal)	3.4 (1.1)	−0.058	−0.194	0.81	3.3 (1.0)	3.5 (1.1)	−1.472	0.142	0.19

*Content in the parentheses is the abbreviation for further analyses. WL, word level; SL, sentence level; TL, text level.

### Data sources and variables

#### Writing composition task

The written samples were collected in class following the instructions from previous writing studies (Wagner et al., 2011; Yan et al., 2012). Children were instructed to write a narrative entitled “An unforgettable day.” Children were expected to write continuously within 10 min, focusing and keeping writing the whole time. For the word the children did not know how to write, they could simply use pinyin to replace it. If the children made a mistake, they could simply cross out the mistaken word and keep writing. If they stopped writing before the 10 min was up, the experimenters encouraged them to continue writing. This task was tested at the whole class level. The written samples were then coded by two raters according to the measurement indices of writing described below.

We used word-level, sentence-level, and text-level measurement indices to evaluate the quality of writing. The word-level measurements were word token and word type, representing the number and density of word in the composition (Wagner et al., 2011). Word token was the total number of words for the writing composition. Word type was the total number of non-repetitive words for the writing composition (Nation, 2001). Word token indicates the fluency, while word type measures the variety of using word to write (Wang, 2017). For example, wǒ xiǎngniàn wǒ de gǒu (我想念我的狗。I miss my dog), there are five types and six tokens in this sentence. For the sentence-level measurement, we used number of premodifiers and mean length of premodifiers as indices of syntactic maturity because language learners use more complex noun phrases when their language levels improve, and these indices are manifested effectively in measuring learners’ syntactic development (Nation, 2001; Biber et al., 2011; Wang, 2017; Wu, 2019). For example, nàgè piàoliɑng de niánqīng nǚshì (那个漂亮的年轻女士, the beautiful young lady), there are three premodifiers before the noun “nǚshì” (lady), which are “nàgè” (the), “piàoliɑng de” (beautiful), and “niánqīng” (young). Mean length of premodifiers were calculated by the mean number of Chinese characters in premodifiers. For the text-level measurement, we used the text content quality as an index. The rating scale of the text content quality was adapted from Kuiken and Vedder (2017)’s functional adequacy scale that composed of five items, including content, task requirements, comprehensibility, coherence, and cohesion. The experimenter was required to rate children’s writing on the five aspects, with the highest score of 6 and the lowest score of 1 for each item. The sum of the scores on the five items was the text content quality of the child. All of the writing indices were rated by two trained experimenters with high inter-rater reliability ranging from 0.768 to 0.999 (Table 1). The criterion-related validity for the content quality measure was 0.744. The averages of the two experimenters were calculated as the measurement indices of writing. As the basic unit of Chinese writing, the total number of characters of the composition was also counted.

#### Reading efficiency task

In order to test the specific relationship between HLE and the writing skills, we included reading efficiency as a control variable. The reading efficiency task followed the procedures of previous studies in alphabetic language (Moll et al., 2009; Wagner et al., 2010). This task consisted of 100 sentences with gradually increasing length. In this task, children were required to silently read 100 sentences and rapidly indicate whether the sentence “makes sense” in 3 min. One example of the reading sentence is “世界上所有的花都是红色的” (All the flowers in the world are red). The total number of characters in correct sentences per minute was calculated as child’s reading fluency score. Therefore, the reading efficiency task used in the present study is scored for reading efficiency, including speed and accuracy. This task was tested at the whole class level. This reading efficiency task has been found to be correlated with both word reading and reading comprehension in previous Chinese studies and it has been suggested to be a reliable proxy with good validity (*r* = 0.746) for reading efficiency in Chinese children (Xue et al., 2013; Su et al., 2017).

#### Home literacy environment questionnaire

Formal literacy experience was measured by an item about the onset age of parent teaching of characters. Specifically, the parents were asked about the age at which they started teaching their child to read characters at home. The choices were 1 = never; 2 = after age 4; 3 = at 3–4 years old; 4 = at 2–3 years old; 5 = at 1–2 years old; and 6 = from 0 to 1 years old. This item has been widely used as a proxy for child’s formal literacy experience in previous studies (Burgess et al., 2002; Su et al., 2017).

Informal literacy experience was measured by seven items about the visiting frequency of various educational locations (science center, art gallery, library, bookstore, aquarium, museum, and tourist attraction). For example, the parents were asked about the frequency they took their children to visit the science center. The choices were 1 = never; 2 = only once; 3 = once every year; 4 = several times every year; 5 = once every month; 6 = once every week, and 7 = several times every week. The average of the seven items was calculated as the index for informal literacy experience. Cronbach’s alpha reliability of the seven items in our sample was 0.80.

The HLE questionnaire was sent to the parents (*via* the children) by the teacher at the same time as children’s behavioral test. Parents were required to finish the questionnaire in 1 day. On the second day, the teacher collected the parent questionnaire at class.

### Analytical method

Statistical analysis was performed in statistical product and service solutions (SPSS) Statistics v.20 (international business machines (IBM) Corporation, Somers, NY, United States). Firstly, descriptive analyses were performed for the writing, reading and HLE measure. Secondly, we compared children’s performance in the five writing measurements and reading efficiency between 3^rd^ graders and 5^th^ graders through independent-sample *t*-tests, effect size of the *t*-tests was calculated by *Cohen’s d*. Thirdly, we performed partial correlations among formal and informal literacy experiences, narrative writing scores and reading efficiency score with the age and sex controlled for 3^rd^ graders and 5^th^ graders respectively. Results of the correlation analyses were corrected for multiple comparisons using the False Discovery Rate (FDR) correction (Benjamini and Hochberg, 1995). In the Results section, we report uncorrected *p*-values and then compare them to the FDR-corrected alpha threshold *q*-value.

The main aim of this study was to assess the predictive pattern of formal and informal literacy experiences for different levels of writing (word, sentence, and text) in Grade 3 and Grade 5. Thus, our main analytical approach was hierarchical linear regression model. As previous studies showed significant association between age, sex, and SES with writing, all analyses were adjusted for the age, sex, and SES variables. Therefore, for each grade, we established a series of hierarchical regression models with age, sex, and SES controlled in the first step, and the formal and informal literacy experiences in the second step. Different indices of writing at each level (i.e., token and type for word level, number of premodifiers and mean length of premodifiers for sentence level, sum of five content quality scores for text level) were looked at separately as dependent variables. In order to highlight the unique contribution of HLE to the writing skills, in the second series of regression models, we included the reading efficiency as a control variable. For each grade, we tested a series of hierarchical regression models with age, sex, and SES controlled in the first step, the reading efficiency in the second step, and the formal and informal literacy experiences in the third step. The dependent variables were narrative writing scores in word, sentence, and text levels.

## Results

In Table 1, *Means*, *standard deviation*, and other descriptive statistic measures of narrative writing, reading, and HLE are reported. Generally, all the measures followed a normal distribution with reasonable skewness and kurtosis. No ceiling effect was found for the writing and reading measures. Considering the grade differences, the numbers of word token [*M_*grade* 3_* = 86.6, *M_*grade* 5_* = 116.8; *t*(248) = −6.254, *p* < 0.001, *Cohens’d* = 0.821], word type [*M_*grade* 3_* = 54.4, *M_*grade* 5_* = 73.2; *t*(248) = −6.472, *p* < 0.001, *Cohens’d* = 0.854], and sentence premodifier [*M_*grade* 3_* = 7.0, *M_*grade* 5_* = 9.3; *t*(248) = −3.346, *p* = 0.001, *Cohens’d* = 0.439] for writing were significant larger in Grade 5 than those in Grade 3. Fifth graders also performed better than 3^rd^ graders on the reading efficiency task [*M_*grade* 3_* = 267.2, *M_*grade* 5_* = 381.0; *t*(248) = −6.380, *p* < 0.001, *Cohens’d* = 0.826]. No significant difference was found in the sentence premodifier mean length [*t*(248) = −0.743, *p* = 0.458, *Cohens’d* = 0.083] and text content quality [*t*(248) = −1.211, *p* = 0.227, *Cohens’d* = 0.158]. Finally, there were no significant differences in the two HLE variables [formal: *t*(248) = −1.735, *p* = 0.084, *Cohens’d* = 0.231; informal: *t*(248) = −1.472, *p* = 0.142, *Cohens’d* = 0.190].

When sex and age were controlled, we found formal literacy experience was correlated with the number of word types (*r* = 0.208, *p* = 0.012 < FDR-corrected *q* = 0.025) and sentence premodifiers (*r* = 0.250, *p* = 0.003 < FDR-corrected *q* = 0.025) for writing in Grade 3, whereas informal literacy experience was correlated with the number of tokens (*r* = 0.369, *p* < 0.001 < FDR-corrected *q* = 0.027), types (*r* = 0.438, *p* < 0.001 < FDR-corrected *q* = 0.027), mean length of premodifiers (*r* = 0.246, *p* = 0.013 < FDR-corrected *q* = 0.027), and content quality (*r* = 0.393, *p* < 0.001 < FDR-corrected *q* = 0.027) for writing in Grade 5 (Table 2). Writing measures were correlated with each other in 3^rd^ grade and 5^th^ grade, except for the correlation between word-level measures and mean length of premodifiers in Grade 3 (token: *r* = 0.147, *p* = 0.078; type: *r* = 0.156, *p* = 0.063). Reading efficiency was correlated with writing measures in Grade 3 (token: *r* = 0.199, *p* = 0.017 < FDR-corrected *q* = 0.025; type: *r* = 0.253, *p* = 0.002 < FDR-corrected *q* = 0.025; premodifier: *r* = 0.194, *p* = 0.020 < FDR-corrected *q* = 0.025; and content quality: *r* = 0.251, *p* = 0.002 < FDR-corrected *q* = 0.025) and was correlated with word-level writing measure in Grade 5 (type: *r* = 0.296, *p* = 0.002 < FDR-corrected *q* = 0.027).

**TABLE 2:** Partial correlations among home literacy environment (HLE), writing, and reading measures controlling for age and sex.

	Formal	Informal	Writing-WL1	Writing-WL2	Writing-SL1	Writing-SL2	Writing-TL	Reading
Formal	−	0.173*	0.165*	**0.208** *	**0.250** **	0.117	0.127	0.077
Informal	0.195*	−	–0.094	–0.034	–0.055	–0.008	–0.009	0.135
Writing-WL1	0.178	**0.369** ***	−	**0.929** ***	**0.559** ***	0.147	**0.536** ***	**0.199** *
Writing-WL2	0.188	**0.438** ***	**0.843** ***	−	**0.559** ***	0.156	**0.537** ***	**0.253** **
Writing-SL1	–0.021	0.191	**0.456** ***	**0.494** ***	−	**0.307** ***	**0.325** ***	**0.194** *
Writing-SL2	0.058	**0.246** *	**0.299** **	**0.462** ***	**0.543** ***	−	**0.246** **	0.172*
Writing-TL	0.197*	**0.393** ***	**0.608** ***	**0.711** ***	**0.401** ***	**0.480** ***	−	**0.251** **
Reading	0.173	0.171	0.213*	**0.296** **	0.021	0.029	0.173	−

Values above the diagonal are correlations of 3^rd^ graders; values below the diagonal are correlations of 5^th^ graders. Formal = onset age of parent teaching, informal = visiting frequency of places, WL1 = word token, WL2 = word type, SL1 = sentence premodifier number, SL2 = sentence premodifier mean length, TL = text content quality. *p < 0.05, **p < 0.01, ***p < 0.001, r values surviving False Discovery Rate (FDR) correction are in bold.

To investigate the influence of formal and informal literacy experiences on the writing skills in word level (token and type), sentence level (number of premodifiers and mean length of premodifiers), and text level (quality of writing content), we performed hierarchical regression analyses for each dependent variable, controlling for sex, age, and SES. *R*^2^ change and standardized β coefficients for each variable are reported in Table 3. We found that girls produced a larger number of word type in Grade 3 (β = 0.165, *p* = 0.045) and Grade 5 (β = 0.284, *p* = 0.001). Girls also produced a larger number of premodifiers relative to boys in Grade 5 (β = 0.211, *p* = 0.036). The formal literacy experience was a significant predictor for the number of token (β = 0.180, *p* = 0.032), type (β = 0.214, *p* = 0.011), and premodifier (β = 0.261, *p* = 0.002) in Grade 3, whereas the informal literacy experience was a significant predictor for the number of token (β = 0.304, *p* = 0.003), type (β = 0.339, *p* < 0.001), premodifier (β = 0.220, *p* = 0.040), mean length of premodifier (β = 0.227, *p* = 0.036), and content quality (β = 0.301, *p* = 0.003) in Grade 5.

**TABLE 3:** Hierarchical regression analyses of writing measures using home literacy environment (HLE) as predictors.

			Word level				Sentence level				Text level	
					
			Token		Type		Premodifier number		Premodifier mean		Content quality	
							
Grade	Steps	Measures	Δ *R*^2^	β	Δ *R*^2^	β	Δ *R*^2^	β	Δ *R*^2^	β	Δ *R*^2^	β
3	1	Control variables	0.044		0.042		0.036		0.023		0.036	
		Age		–0.051		–0.041		–0.076		–0.066		0.017
		Sex		0.147		0.165*		0.109		0.125		0.159
		SES		–0.106		–0.082		–0.110		–0.029		0.120
	2	Home literacy environment	0.032*		0.044*		0.065*		0.014		0.021	
		Formal		0.180*		0.214*		0.261**		0.120		0.137
		Informal		–0.077		–0.033		–0.051		–0.015		–0.086

5	1	Control variables	0.100*		0.192***		0.041		0.022		0.126**	
		Age		0.001		–0.002		0.030		0.025		–0.049
		Sex		0.184		0.284**		0.211*		0.021		0.122
		SES		0.114		0.186		–0.059		0.054		0.194
	2	Home literacy environment	0.091**		0.107***		0.041		0.044		0.090***	
		Formal		0.090		0.071		–0.050		0.001		0.092
		Informal		0.304**		0.339***		0.220*		0.227*		0.301**

Formal = onset age of parent teaching, informal = visiting frequency of places. *p < 0.05, **p < 0.01, ***p < 0.001.

In an extended model, we performed hierarchical regression analyses controlling the influence of reading on writing. In the model, we entered reading efficiency variable before the HLE variables. *R*^2^ change and standardized β coefficients for each variable are reported in Table 4. We found that girls produced a larger number of word types and sentence premodifiers relative to boys in Grade 5 (β = 0.280, *p* = 0.001; β = 0.211, *p* = 0.037). The reading efficiency predicted the number of word token (β = 0.219, *p* = 0.008), word type (β = 0.267, *p* = 0.001), sentence premodifier (β = 0.202, *p* = 0.014), mean length of premodifier (β = 0.180, *p* = 0.035), and text content quality (β = 0.240, *p* = 0.004) in Grade 3. The effect of HLE on the writing skills remained after controlling the influence of reading efficiency on writing. Specifically, the formal literacy experience was a significant predictor for the number of word token (β = 0.168, *p* = 0.042), word type (β = 0.199, *p* = 0.014), and sentence premodifier (β = 0.250, *p* = 0.002) in Grade 3, whereas informal literacy experience was a significant predictor for the number of word token (β = 0.282, *p* = 0.005), word type (β = 0.315, *p* = 0.001), sentence premodifier (β = 0.224, *p* = 0.039), mean length of premodifier (β = 0.236, *p* = 0.031), and text content quality (β = 0.294, *p* = 0.004) in Grade 5.

**TABLE 4:** Hierarchical regression analyses of writing measures using reading efficiency and home literacy environment (HLE) as predictors.

			Word level				Sentence level				Text level	
					
			Token		Type		Premodifier number		Premodifier mean		Content quality	
							
Grade	Steps	Measures	Δ *R*^2^	β	Δ *R*^2^	β	Δ *R*^2^	β	Δ *R*^2^	β	Δ *R*^2^	β
3	1	Control variables	0.044		0.042		0.036		0.023		0.036	
		Age		–0.060		–0.052		–0.085		–0.074		0.007
		Sex		0.121		0.133		0.085		0.104		0.131
		SES		–0.139		–0.123		–0.140		–0.056		0.084
	2	Reading efficiency	0.048**		0.073**		0.044*		0.033		0.056**	
		Sentence reading efficiency		0.219**		0.267**		0.202*		0.180*		0.240**
	3	Home literacy environment	0.030**		0.038**		0.060**		0.011		0.019*	
		Formal		0.168*		0.199*		0.250**		0.109		0.123
		Informal		–0.092		–0.052		–0.065		–0.028		–0.103

5	1	Control variables	0.100*		0.192***		0.041		0.022		0.126**	
		Age		–0.008		–0.012		0.032		0.029		–0.051
		Sex		0.180		0.280**		0.211*		0.023		0.120
		SES		0.116		0.189*		–0.059		0.053		0.195
	2	Reading efficiency	0.043**		0.049***		< 0.001		0.001		0.010**	
		Sentence reading efficiency		0.162		0.174*		–0.033		–0.067		0.050
	3	Home literacy environment	0.073**		0.087***		0.042		0.047		0.082***	
		Formal		0.068		0.047		–0.045		0.010		0.085
		Informal		0.282**		0.315**		0.224*		0.236*		0.294**

Formal = onset age of parent teaching, informal = visiting frequency of places. *p < 0.05, **p < 0.01, ***p < 0.001.

## Discussion

The present study aims to assess Chinese children’s writing development according to different levels of language (word, sentence, and text) and investigate the influence of HLE on children’s writing development. Results showed that children’s writing abilities differed between Grade 3 and Grade 5 on the word and sentence levels, while no significant difference was found in the writing of text level. Different aspects of HLE played different roles in the development of writing. More specifically, formal literacy experience indexed by the onset age of parent teaching predicted the writing performance (word and sentence levels) in Grade 3, while informal literacy experience indexed by visit of various educational locations predicted the writing performance (word, sentence, and text levels) in Grade 5. After controlling the effect of age, sex, SES, and reading efficiency on the writing skills, the prediction pattern of HLE on writing performance remained, indicating the unique effect of HLE on writing composition.

Compared with ample studies in alphabetic languages, studies in Chinese writing development were relatively few and limited to the handwriting and spelling of writing (Tan et al., 2005; Guan et al., 2011). The present study investigated the developmental characteristics of Chinese writing composition on different levels of language. In line with previous studies in alphabetic languages (Whitaker et al., 1994; Puranik et al., 2008; Koutsoftas, 2018), the present study found various developmental characteristics of the writing at different levels of language. On the word and sentence levels of writing, 5^th^ graders performed better than the 3^rd^ graders, which was consistent with findings in a previous study of alphabetic language (Puranik et al., 2008). Regarding the writing of text level, no significant difference was found between 3^rd^ and 5^th^ graders, which was also similar with a previous study reporting no differences on the text level of writing among 4^th^, 5^th^, and 6^th^ graders (Whitaker et al., 1994). The similar developmental pattern between Chinese and alphabetic languages may indicate the universal property of writing development across different orthographies. Considering the educational implications of these findings, absent development on the text level of writing may suggest that the emphasis of writing instruction in intermediate grades should be focused on the text level of writing. For instance, the measures (content, task requirements, comprehensibility, coherence, and cohesion) assessed writing quality on the text level in the present study may be important teaching directions for the educators.

Previous studies have found the relationship between HLE and reading development (Levy et al., 2006; Inoue et al., 2018; Georgiou et al., 2021). The present study found the association between HLE and writing narratives, which verify the tight relationship between reading and writing and extend the home literacy model to the writing composition process. In the present study, we found that formal literacy experience indexed by the onset age of parent teaching predicted the writing in word level (number of word) and sentence level (number of premodifiers) in Grade 3. More specifically, the earlier the children learned character, the larger number of word and premodifiers they produced in the writing process. Early access to Chinese characters promoted the development of early linguistic skills, like orthographic, phonological, morphological, and vocabulary skills (Su et al., 2017). These skills may subsequently enhance children’s literacy skills like recognizing and writing more word, especially including the word used as premodifiers in better expressing the sentence. The educational implication of this finding may be the importance of early parent teaching, especially on the characters teaching of Chinese language.

Another interesting finding of the present study is that the informal literacy experience indexed by visit of various educational locations (science center, art gallery, bookstore, aquarium, museum, and tourist attraction) is related to all levels of writing performance in Grade 5. One superordinate knowledge important for writing is domain knowledge about content, which includes what is often referred to as “world knowledge” (the knowledge a reader brings to a text) (Fitzgerald and Shanahan, 2000). The increased vocabularies of place names, activities, and terminologies due to visit might explain the richness of words used in composition and increased world knowledge due to various visiting experience might explain the increased content quality of writing (Dunsmuir and Blatchford, 2004; Kim et al., 2011; Wang, 2017). The present study showed that children’s visit to various places such as a museum, library, or bookstore with parents benefits their writing development in upper grade. However, as children grow up, family members seem to lose much of their out-home visit due to increasing work and excessive use of electronic products (e.g., smartphone and tablet computer). Therefore, educators should advocate the importance of visiting outside home on the writing process, especially for parents of upper graders.

Finally, the work presented here had several limitations. Firstly, although we selected the onset age of parent teaching as the variable for formal literacy experience and visiting frequency of various places as the variable for informal literacy experience, more concrete formal (e.g., actual frequency and time of children’s literacy-related behavior at home, duration of the parent teaching behavior) and informal literacy experiences (e.g., quantity and quality of parent-child literacy-related activity during visiting outside home) should be explored. Secondly, there may be mediators between HLE and writing skills. For instance, vocabulary and word knowledge may be important in explaining the relationship between HLE and writing. However, we did not explicitly measure children’s vocabulary and word knowledge in the current study. Further study should measure them explicitly and explore the relationship between HLE, vocabulary, word knowledge and writing. Finally, this study was a cross-sectional study with only 3^rd^ and 5^th^ graders, future research should combine longitudinal methodology with careful examination on the effect of literacy experience at home, school and social situation in children’s composition writing.

## Data availability statement

The raw data supporting the conclusions of this article will be made available by the authors, without undue reservation.

## Ethics statement

The studies involving human participants were reviewed and approved by the College of Elementary Education, School of Psychology, Capital Normal University. Written informed consent to participate in this study was provided by the participants’ legal guardian/next of kin.

## Author contributions

MS, YF, and BQ performed material preparation, data collection, and analysis. MS and WZ wrote the first draft of the manuscript. JW and WZ commented on previous versions of the manuscript. All authors contributed to the study conception and design, and read and approved the final manuscript.
